# Mohs micrographic surgery for cutaneous adnexal carcinoma with squamous metaplasia and subsequent identification of metastasis: A case report

**DOI:** 10.1016/j.jdcr.2025.09.025

**Published:** 2025-10-10

**Authors:** Katayoun Khalilian, Alec K. Gramann, Travis Vandergriff, Rajiv I. Nijhawan, Divya Srivastava

**Affiliations:** Department of Dermatology, University of Texas Southwestern Medical Center, Dallas, Texas

**Keywords:** metastatic adnexal carcinoma, Mohs micrographic surgery, poorly differentiated adnexal carcinoma, surgical dermatology

## Introduction

Adnexal carcinomas are rare cutaneous malignant neoplasms arising from adnexal structures of the epidermis and subcutis^1^ that most commonly present as primary tumors typically retaining features of the cells and structures from which they originate.^1^ Adnexal neoplasms may display multiple lines of differentiation and rarely can present as metastatic disease with poor differentiation or histologic features of multiple adnexal structures.^1^, ^2^, ^3^ Surgical excision, either with wide local excision or Mohs micrographic surgery (MMS), is the most frequent modality for treatment of these cutaneous neoplasms and can provide diagnostic clues to indicate if additional metastatic evaluation is warranted.^1^^,^^4^, ^5^, ^6^ Here, we present a case of poorly differentiated adnexal carcinoma with squamous features of the scalp treated with MMS, which was subsequently determined to be associated with metastasis.

## Case report

**Fig 1:**
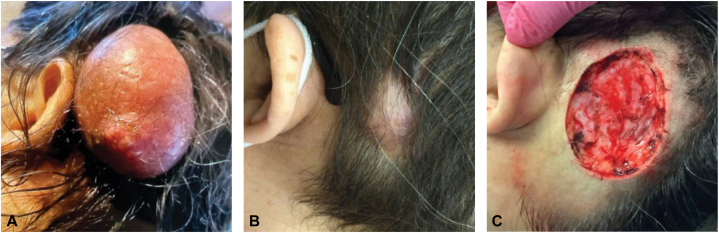
Clinical images of adnexal carcinoma of the scalp. **(A)** Mass prior to surgical intervention showing an exophytic smooth mass on the postauricular scalp. **(B)** Initial presentation pre-Mohs micrographic surgery showing an alopecic patch at the site of previous excision with subcutaneous nodularity on examination. **(C)** Defect following Mohs micrographic surgery with clear margins after two stages.

A 54-year-old woman presented to an outside facility for evaluation of a growth on her scalp (Fig 1, *A*). The lesion was reported to be present and stable in size for over 10 years, with recent rapid growth over six months prior to presentation. The lesion had notable keratinaceous and caseous discharge, clinically consistent with an epidermoid cyst. She underwent excision with plastic surgery. Pathology showed an infiltrating adnexal carcinoma confirmed with immunohistochemistry with mixed differentiation, with the tumor extending to the margins. She was referred for MMS for further treatment.

On evaluation, the patient was noted to have a 6.0 × 3.0 cm plaque of scar and alopecia, consistent with recent excision (Fig 1, *B*). Notably, subcutaneous nodularity was palpable beyond the apparent margin of the excision, indicating a likely local residual tumor. No palpable occipital or cervical lymphadenopathy was noted on initial examination with dermatology. After discussion of treatment options with the patient, she opted to undergo MMS with planned reconstruction by plastic surgery following excision. Clinically apparent residual lesion as identified by subcutaneous nodularity was debulked and sent for permanent section for further analysis. The initial stage of MMS revealed poorly differentiated sheets of atypical cells extending to the peripheral and deep margins. The lesion was cleared in the second stage. Final defect size following clearance in the second stage was 9.0 × 6.0 cm (Fig 1, *C*).

**Fig 2:**
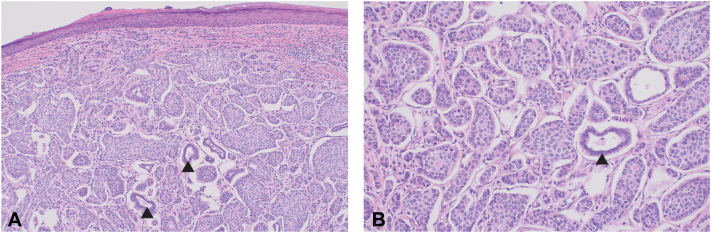
Histologic images from Mohs micrographic surgery debulk, confirming the diagnosis of adnexal carcinoma. **(A)** Malignant gland-forming adnexal epithelial neoplasm in the reticular dermis without clear connection to overlying epidermis. **(B)** Notable cytologic atypia and infiltrative growth of a glandular epithelial neoplasm. Arrowheads, glandular structures localized to the reticular dermis. (Original magnifications: **A,** ×20; **B,** ×100.)

**Fig 3:**
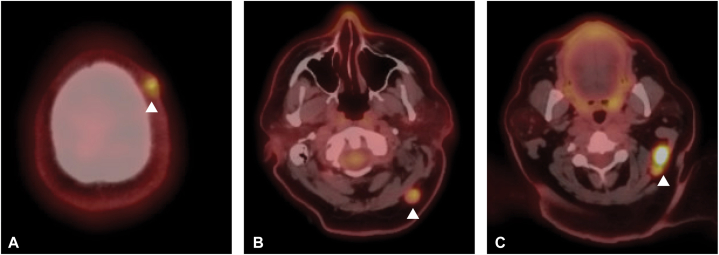
PET/CT imaging of the head and neck shows FDG-avid lymphadenopathy of the postauricular and occipital nodes, and an incidental FDG-avid lesion (arrowheads). **(A)** Incidental identification of a secondary scalp lesion, suspected to be unrelated to a proliferating trichilemmal tumor. **(B)** FDG-avid occipital node positive for adnexal carcinoma on lymph node dissection. **(C)** FDG-avid postauricular node positive for adnexal carcinoma on lymph node dissection.

**Fig 4:**
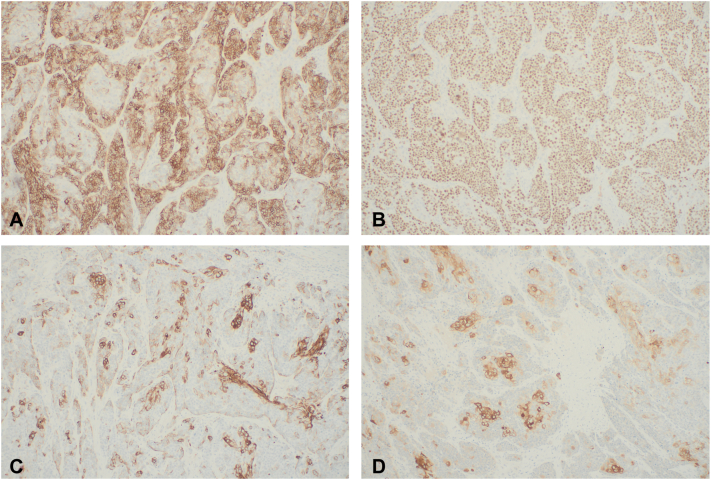
Immunohistochemistry images from Mohs micrographic surgery debulk, confirming the diagnosis of adnexal carcinoma. **(A)** Diffuse EMA expression. **(B)** Diffuse p63 nuclear expression. **(C)** Patchy CK903 expression. **(D)** Focal expression of AE1AE3. (Original magnifications: **A,** ×100; **B,** ×100; **C,** ×100; **D,** ×100.)

Permanent section processing of the debulk specimen confirmed the presence of tumor cells in the reticular dermis with squamous and basaloid epithelial differentiation in nests, strands, and sheets (Fig 2). Immunohistochemistry identified diffuse p63 and EMA expression, patchy CK903 expression, and focal expression of AE1AE3 and GATA3 (Fig 3). A diagnosis of poorly differentiated adnexal carcinoma with focal squamous differentiation was made. Given the deep-seated nature of the tumor and the lack of classic uniform features of adnexal differentiation frequently observed in primary cutaneous adnexal carcinomas, there was suspicion that this lesion could represent cutaneous metastasis. The patient was referred for further evaluation with medical oncology and radiation oncology. She subsequently underwent positron emmission tomography/compute tomography (PET/CT), which identified additional fluorodeoxyglucose (FDG)-avid lesions, including left cervical lymph nodes concerning for nodal metastases (Fig 4). Incidentally, the patient was found to have an FDG-avid uterine lesion that was ultimately determined to be an unrelated low-grade primary endometrial carcinoma and a second scalp lesion ultimately found to be an unrelated proliferating trichilemmal tumor. No additional sites concerning a primary carcinoma of a visceral organ were identified, suggesting that her cutaneous disease was the primary tumor. Dissection of the left side of the neck and retroauricular lymph nodes by otolaryngology confirmed the presence of poorly differentiated carcinoma in 12 of 93 identified lymph nodes, with subcapsular extension noted in multiple nodes. Because of the extent of disease, adjuvant chemoradiotherapy with carboplatin and paclitaxel was initiated. Progression was identified on subsequent scans, and patient was transitioned to pembrolizumab. The patient continued to have progression of disease while on immunotherapy and expired shortly after initiating treatment.

## Discussion

Adnexal carcinomas represent a rare entity of cutaneous malignant neoplasms, arising from adnexal structures within the skin.^1^^,^^4^ Although many arise *de novo*, a preceding benign adnexal neoplasm is occasionally observed. Often, these tumors maintain features consistent with their structure of origin. However, these tumors can present with mixed structural lineage and, in rare circumstances, present without clear features of the structure of origin, as in the case presented here, where histology is consistent with adnexal carcinoma with features of squamous metaplasia.^3^^,^^7^ Notably, Surveillance, Epidemiology, and End Results ICD-O-3 coding manual includes specific codes for adenocarcinoma with various types of metaplasia, including squamous, osseous and cartilage, neuroendocrine, spindle cell, and apocrine. In our review of Surveillance, Epidemiology, and End Result data, 5603 cases of adnexal carcinoma of the skin have been reported. Of those, two cases of skin adnexal carcinomas with features of squamous metaplasia were identified, with neither case identified in the analysis of Surveillance, Epidemiology, and End Result data being associated with metastatic disease as seen in the case presented here. Previous studies of adnexal carcinomas of the skin have shown relatively low rates of metastatic disease, but have shown that the presence of metastatic disease is associated with worse overall and disease-specific survival.^5^^,^^6^^,^^8^ However, due to the rarity of these cases, the association between tumor differentiation status or associated metaplastic features and the risk of metastasis, as well as overall and disease-specific survival, is unclear.

With the rarity of adnexal carcinomas within the population, and even further rarity of tumor subtypes involving metaplasia, it is unclear what modalities of evaluation and treatment are most appropriate for patients with poorly differentiated neoplasms. In cases of more differentiated cutaneous adnexal carcinomas, early evaluation for surgical intervention is important because it is often the preferred modality of treatment.^2^^,^^5^, ^6^, ^7^, ^8^, ^9^ Some reports show resection with MMS has more favorable surgical outcomes and lower rates of recurrence.^10^ As frequently observed in other malignancies, metaplasia and dedifferentiation can be indicators of greater risk of metastatic or aggressive disease. In cases where systemic therapy is deemed necessary, immunotherapy alone or in combination with chemotherapy has shown benefit in some cases.^11^, ^12^, ^13^ Based on our experience with this case, the presence of metaplastic or dedifferentiated features within cutaneous adnexal carcinomas should increase suspicion for nodal or distant metastatic disease, even in cases where there are no clear clinical signs of metastasis, and further evaluation with additional imaging and workup by medical oncology may be appropriate.

## Conflicts of interest

None disclosed.
